# Finite-time robust speed control of synchronous reluctance motor using disturbance rejection sliding mode control with advanced reaching law

**DOI:** 10.1371/journal.pone.0291042

**Published:** 2023-09-11

**Authors:** Usman Nasim, Abdul Rauf Bhatti, Muhammad Farhan, Akhtar Rasool, Arslan Dawood Butt

**Affiliations:** 1 Department of Electrical Engineering and Technology, Government College University, Faisalabad, Punjab, Pakistan; 2 Department of Computer Science, COMSATS University Islamabad, Sahiwal Campus, Sahiwal, Punjab, Pakistan; 3 Department of Electrical Engineering, University of Botswana, Gaborone, Botswana; University of Hull, UNITED KINGDOM

## Abstract

In recent years, there has been a significant focus on synchronous reluctance motors (SynRM) owing to their impressive efficiency and absence of magnetic material. Although the SynRM shows great potential for use in electric vehicles, its widespread adoption is limited by unmodeled dynamics and external disturbances. Moreover, the uncertainty factor significantly restricts SynRM’s peak efficiency and superior control performance, leading to an unjustifiable current loop reference command. To address these issues, this work presents various new research contributions which focus on the robust control of SynRM to optimize performance through the novel reaching law-based sliding mode control. Initially, a novel advanced sliding mode control reaching law (ASMCRL) with adaptive gain is proposed, to enhance the acceleration of the system state reaching the sliding surface. After that, an extended state observer (ESO) is designed to estimate and compensate for the overall disturbances of the system. Finally, the ASMCRL and ESO are integrated to design two nonlinear controllers namely, the disturbance-rejection sliding mode controller (DRSMC) and the disturbance-rejection sliding mode speed regulator (DRSMSR) for SynRM. The proposed DRSMSR eliminates the steady-state error and eradicates inherent chattering in DRSMC. Moreover, this yields a system trajectory that converges to a predetermined proximity of the sliding surface, irrespective of any lumped disturbances. The steady-state error of DRSMSR is less as compared to DRSMC. Furthermore, the speed response of this technique is 22.62% faster as compared to the state-of-the-art finite-time adaptive terminal sliding mode control. Additionally, the asymptotic stability of the proposed system is validated using Lyapunov’s theorem. Thus the experimental results demonstrate the effectiveness and robustness of the proposed approach.

## Introduction

Electric motors are widely used in various industrial sectors such as manufacturing, transportation, energy production, and many others [1]. Permanent magnet synchronous motors (PMSM), induction motors (IM), and synchronous reluctance motors (SynRM) are commonly used electric motors in industrial applications. PMSMs are constructed using permanent magnets, which are usually composed of rare-earth metals, such as neodymium-iron-boron alloy (NdFeB) [2]. The significant increase in the price of rare earth metals has led to a widespread search for alternatives to PMSM including machines with less magnetic material or those that operate without magnets [3]. Another drawback of PMSM is its susceptibility to short-circuit faults [4]. Furthermore, in high-speed applications, PMSM is sensitive to temperature changes [5].

The SynRM has emerged as a promising alternative to PMSM [6], owing to its advantages such as a simple rotor structure with no copper losses, absence of magnetic materials, high performance, and low cost [7]. However, SynRM despite its advantages has certain limitations in terms of non-linearity, uncertainty, and parameter variations [8]. The drive system of a SynRM is affected by significant nonlinear uncertainties resulting from factors such as air-gap field harmonics, the impact of flux saturation, and cogging torque [9]. Numerous techniques have been developed for controlling the speed of the SynRM, including intelligent, linear, and nonlinear methods [10].

A direct torque control (DTC) based SynRM drive system is presented in [11]. Predictive control of SynRM utilizing an extended Kalman filter is proposed in [12]. In [13], the control of SynRM has been achieved through the utilization of model predictive control with online parameter estimation. Neural network-based control of SynRM has been proposed in [14]. Although the current methods have made significant contributions toward optimal speed and position control, the compensation for uncertainties and disturbances in the system under various conditions is a demanding issue that needs to be resolved.

Sliding mode control (SMC) is a robust nonlinear control technique that can address the issue of uncertainties caused by factors such as vague and time-varying load torque, unknown initial rotor position angle, and uncertain motor parameters [15]. SMC has been applied in many electrical and mechanical control systems [16], and has been utilized in motor drives for many years [17]. Researchers have made significant efforts to tackle the challenges of deteriorated drive system performance caused by disturbances through the use of SMC [18].

The study presented in [19] employed SMC for robust speed control of SynRM, a modified second-order SMC combined with the radial basis function has been utilized for precise speed control of SynRM. However, the speed response is slow and error increases with the external load increase. In [20], a finite time terminal SMC for SynRM has been proposed, which has a robust control response against parameter variations. An observer is designed to overcome the disturbances, and a new-reaching law is proposed to reduce chattering. Although the proposed method has superior performance compared to conventional terminal SMC, the speed response shows high ripples and the maximum speed error is also high. A complementary SMC for SynRM has been proposed in [21], with a new d-axis current control technique that achieves variable d-axis current instead of constant. This approach yields a good steady-state response, but the speed error increases as the load torque increases.

The focus of our research work is to address the issues of uncertainty, parameter variations, and load torque fluctuations in SynRM by using a disturbance-rejection sliding mode controller (DRSMC). To mitigate steady-state errors, the disturbance-rejection sliding mode speed regulator (DRSMSR) using integral sliding mode control (ISMC) has also been proposed. The selection of the SMC technique is based on its robustness and accuracy, as highlighted in [22]. SMC is a robust solution against disturbances and parameter variations [23]. Identifying an appropriate sliding surface and designing a reaching law are important components of SMC [24]. However, the conventional SMC method is known to suffer from chattering [25]. The design of appropriate reaching law and selection of suitable sliding surface can resolve the chattering issue [26]. To address the issue of chattering, a novel advanced sliding mode control reaching law (ASMCRL) has been introduced, which incorporates the typical exponential reaching law (TERL) and a terminal attractor. The adaptive gain ASMCRL is designed to ensure that the system state reaches the sliding manifold in a finite time without causing chattering. Additionally, a nonlinear extended state observer (ESO) is developed to eliminate the lumped disturbances from the output effectively. This ESO is used as the feed-forward compensation component to be integrated into the speed controller.

The main contributions of the paper are as follows:

1. Developing an advanced reaching law for the SynRM drive system that significantly reduces the time taken to reach the sliding surface and overcomes the issue of chattering, which is a major limitation of sliding mode control.
2. An extended state observer is designed to address the issue of unmodeled dynamics and disturbances. This observer is not dependent on motor parameters, making it independent and efficient in resolving the problem.
3. To combine the ASMCRL and ESO in the sliding mode and integral sliding mode control of SynRM for speed regulation. This combination has shown promising results as it effectively reduces chattering while accelerating the convergence speed. As a result, the maximum speed error is minimized, and steady-state ripples are eliminated.

The organization of the rest of the article is as follows. The next section provides the block diagram and description of the proposed control system. The design of the sliding mode control reaching law presents the complete proposed ASMCRL. The mathematical model of SynRM and the process for designing the overall observer-based speed controllers are discussed in the section SynRM controller design. In simulation verification, the performance evaluation of ASMCRL, DRSMC, and DRSMSR is presented along with the results. Finally, the conclusion section concludes the article.

## Description of the proposed system

**Fig 1:**
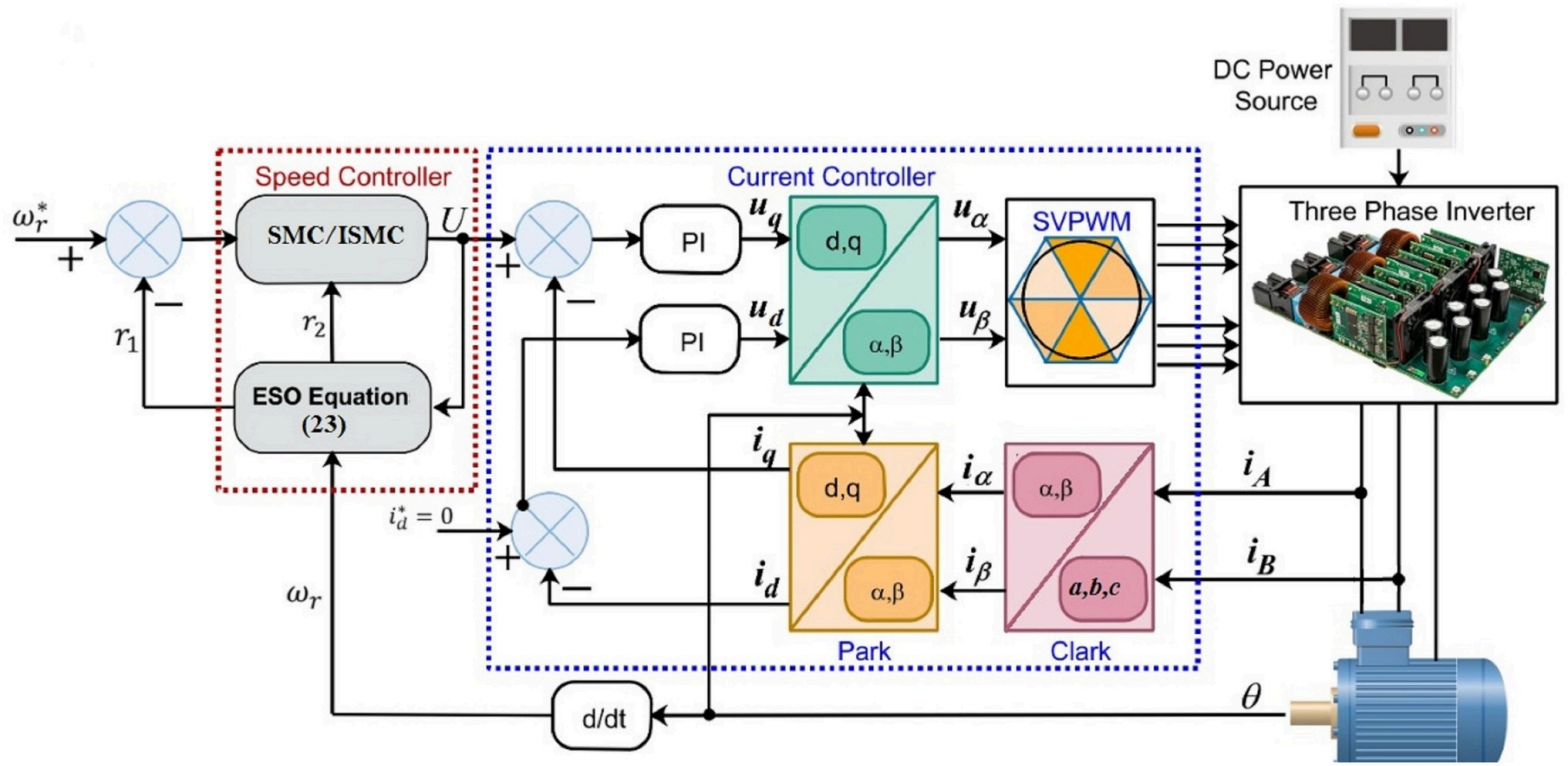
Block diagram of SynRM speed-regulation system.

The complete system description for the robust speed control of SynRM is shown in Fig 1. The system has a speed controller and a current controller. Reference speed ω* is the desired speed at which SynRM is required to be operated. The speed controller is comprised of an ESO-based sliding mode controller with proposed reaching law. Initially, the proposed DRSMC-based speed controller with advanced reaching law is used in the speed controller. Then to reduce the steady-state error, the proposed DRSMSR-based speed controller with advanced reaching law is used. The current controller is based on the PI controllers. Two PI controllers have been used for the d-axis and q-axis current control respectively. The DRSMSR generates $i_{q}^{*}$ as the control input *U*, which is fed forward to the current controller block and observer. The ESO estimates the load disturbances and it also observes the uncertainties. The three-phase current is converted into the two-phase stationary reference frame using the Clark transformation. Rotor position *θ* is an important parameter, it is used in Park transformation for the reference frame conversion from the two-phase stationary reference frame to the two-phase synchronously rotating reference frame. The SynRM is fed with a space vector pulse width modulation (SVPWM) inverter.

### Mathematical model of SynRM

The permanent magnet and field winding are not required in the rotor of SynRM, so it has high efficiency, ruggedness, and cost-effectiveness. The current equations of SynRM in the d-q synchronous reference frame are represented below [27],

$$\begin{array}{cc} \frac{di_{ds}}{dt}\; & =\frac{1}{L_{d}}\;\left(v_{ds}-R_{s}i_{ds}+\omega_{e}L_{q}i_{qs}\right) \end{array}$$ (1)

$$\begin{array}{cc} \frac{di_{qs}}{dt}\; & =\frac{1}{L_{q}}\;\left(v_{qs}-R_{s}i_{qs}-\omega_{e}L_{d}i_{ds}\right) \end{array}$$ (2)

where *i*_*ds*_ is the d-axis equivalent current and *i*_*qs*_ is the q-axis equivalent current in synchronous reference frame. The d-axis voltages *v*_*ds*_, the q-axis voltages *v*_*qs*_, stator resistance *R*_*s*_, d-axis self-inductance *L*_*d*_, q-axis self-inductance *L*_*q*_ and electrical angular velocity *ω*_*e*_ are used. In this research work, the *d*−axis current is fixed as constant while the *q*−axis current has been controlled using the sliding mode control technique. The nomenclature of all variables is also provided in Table 1 at the end of the article.

**Table 1:** Nomenclature.

Symbol	Meaning
*c*_1_, *c*_2_	Positive value constants
*a*_1_, *a*_2_, *k*_1_, *k*_2_	Design parameters with constant positive value
*e*_1_, *e*_2_	Error terms
*s*	Sliding surface
*x*_1_(*t*), *x*_2_(*t*)	State variables
*i*_*ds*_, *i*_*qs*_	d-axis and q-axis current
*v*_*ds*_, *v*_*qs*_	d-axis and q-axis voltage
*L*_*d*_, *L*_*q*_	d-axis and q-axis self inductance
*R*_*s*_	Stator resistance
*J*, *B*	Inertia coefficient, damping coefficient
*P*	Number of poles
*T*_*e*_, *T*_*L*_	Electromagnetic torque, Load Torque
*Q*, *Q*_1_, *Q*_2_	Positive elements that determines the control gains
*U*	Control input q-axis reference current
*θ*_*r*_	Mechanical position of rotor
*α*, *β*	Positive constant between 0 and 1
Δ*ψ*, Δ*B*_*r*_, Δ*δ*	Disturbance and parameter variations
*ω*_*e*_, *ω*_*r*_	Electrical speed, mechanical speed
*γ*	Estimated parameter uncertainty
*σ*	Load disturbance observed by ESO
*ϵ*_1_(*t*)	Error between observed and actual speed
*ρ*_1_, *ρ*_2_, *ρ*_3_	Observer gains of proposed technique

The mechanical speed of the rotor, current rotor position, and electrical speed equations of SynRM are written as follows [28],

$$\begin{array}{cc} \frac{{d\omega }_{r}}{dt}\; & =\frac{1}{J}\;\left(T_{e}-T_{L}-B\omega_{r}\right) \end{array}$$ (3)

$$\begin{array}{cc} \frac{d\theta_{r}}{dt} & =\omega_{r} \end{array}$$ (4)

$$\begin{array}{cc} \omega_{e}\; & =\frac{P}{2}\omega_{r} \end{array}$$ (5)

where *T*_*e*_ represents electromagnetic torque, *T*_*L*_ represents load torque, *B* represents the coefficient of damping, *ω*_*r*_ is the mechanical angular velocity, *θ*_*r*_ is the rotor position, *P* is number of poles and *J* is moment of inertia.

The electromagnetic torque is expressed as,

$$\begin{array}{c} T_{e}=\frac{3}{2}\frac{P}{2}\left(L_{d}-L_{q}\right)i_{ds}i_{qs} \end{array}$$ (6)

It is clear from (6), the electromagnetic torque can be controlled by using *i*_*ds*_, *i*_*qs*_ or combination of both. A 6-pole SynRM has been used in this work and the parameters of the SynRM are provided in Table 2. The SynRM with high saliency *L*_*d*_/*L*_*q*_ ratio has a high power factor and high torque density, so the value of *L*_*d*_ is higher as compared to *L*_*q*_.

**Table 2:** Parameters of SynRM.

Motor Parameter, symbol	Value	unit
Coefficient of inertia, J	0.0008	kg.m^2^
Coefficient of damping, B	0.0003	N.m.s/rad
Number of poles, P	6	poles
Stator resistance, *R*_*s*_	1.55	*Ω*
d-axis inductance, *L*_*d*_	116	mH
q-axis inductance, *L*_*q*_	18	mH

## Design of sliding mode control reaching law

**Fig 2:**
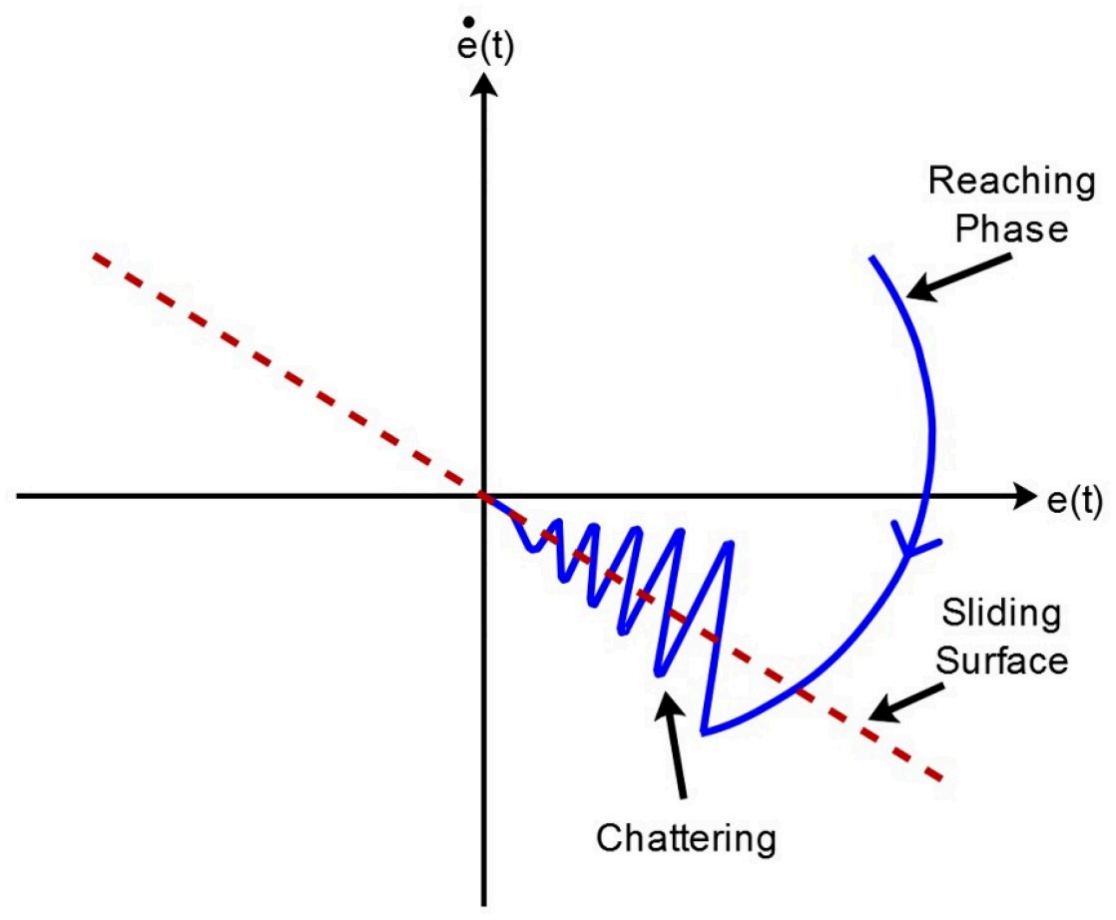
Structure of sliding mode control.

The selection of a sliding surface that provides the required performance is the first step in sliding mode control. The second step is to design the reaching law, which pushes the system states to reach and stay along the sliding surface [29]. The design of an appropriate reaching law accelerates and enhances the quality of the reaching phase response, hence it reduces the overall system response time [30]. Once the sliding manifold is attained, the velocity of reaching decreases to zero, and the system state is converged on the sliding surface [31]. In Fig 2, both portions of SMC i.e., the reaching phase and the sliding phase are shown.

In the case of constant rate reaching law, reaching time is infinite. To reduce the arrival time, the intimation reach term was introduced in constant along with the proportional rate or typical exponential reaching law (TERL) [32], as shown below.

$$\begin{array}{c} \frac{ds}{dt}=-Q_{1}s-Q_{2}sgn\left(s\right),\;\;Q_{1}>0,Q_{2}>0 \end{array}$$ (7)

Where *sgn*(*s*) is the signum function, *Q*_2_*sgn*(*s*) is the constant term, and *Q*_1_*s* represents the proportional reach term. With a proper selection of parameters, the reaching law is used to get the control law [33]. TERL resolves the access issue, but the speed of reaching the sliding surface is dependent on the parameter *Q*_1_. The high value of *Q*_1_ increases the reaching speed, however, the chattering also increases.

For the positive value of *s*, (7) is represented as.

$$\begin{array}{c} \frac{ds}{dt}=-Q_{1}s-Q_{2} \end{array}$$ (8)

The value of reaching time is attained by taking the integration of (7) from 0 to t and *s*(*t*) = 0.

$$\begin{array}{c} t^{*}=\frac{1}{Q_{1}}[ln\{\frac{Q_{2}}{Q_{1}}+s\left(0\right)\}-ln\frac{Q_{2}}{Q_{1}}] \end{array}$$ (9)

It is obvious from (9) that constant value *c* and reaching time are inversely proportional. The reaching time decreases and reaching velocity increases if the value of the constant is increased and vice versa. Therefore, in order to get fast dynamic performance, a higher constant value is required. In this case, the reaching speed will also be high near the sliding surface, so chattering will be increased. Hence the fixed value of the index term creates the problem of high chattering. This problem is considered and solved by the proposed advanced reaching law.

### Proposed ASMCRL

The TERL is utilized to propose the ASMCRL, proposed reaching law updates according to system state and fluctuations. Appraising that terminal attractor |*s*|^*β*^*sgn*(*s*) term smoothness near the sliding manifold [20], the ASMCRL is given as

$$\left\{\begin{array}{l} \frac{ds}{dt}=-c_{1}|s{|}^{\alpha sgn\left(|s|-1\right)}s\;-\;\frac{c_{2}|s{|}^{\beta }sgn\;\left(s\right)}{\left(1+Q|x|\right)}\\ \underset{x\to \infty }{\text{lim}}|x|=0,Q>0,c_{1}>0,\;c_{2}>0,\;0<\alpha <1,0<\beta <1 \end{array}\right.$$ (10)

The advanced reaching law utilizes the norm of system state *x*. The speed response of the system at different stages, distant from the sliding surface and near the equilibrium point, is regulated by advanced reaching law. In the beginning, the system state is distant from the sliding manifold i.e. *s* > 1 and |*x*|>1, so

$$\begin{array}{c} sgn(|s|-1)\;=\;1 \end{array}$$ (11)

$$\begin{array}{c} \frac{c_{2}}{\left(1+Q|x|\right)}\;\ll c_{2} \end{array}$$ (12)

From both (11) and (12), it is obvious that the convergence speed is much greater than the TERL in (7). When the system is close to sliding surface |*s*|<1,

$$\begin{array}{c} \frac{c_{2}}{\left(1+Q|x|\right)}\;<\;c_{2} \end{array}$$ (13)

$$\begin{array}{c} sgn(|s|-1)\;=-1 \end{array}$$ (14)

*c*_1_|*s*|^−*α*^*s* < *c*_1_|*s*|^*α*^*s* hence, the proposed reaching law is slick upon reaching the sliding surface. Finally, when the system is nearly zero, (10) becomes

$$\begin{array}{c} \frac{ds}{dt}=-c_{1}s-c_{2}{\left|s\right|}^{\beta }sgn\;\left(s\right) \end{array}$$ (15)

So, the ASMCRL is reduced to a smoothing effect. The time required from an initial state to a sliding surface is finite.

$$\begin{array}{c} t=\frac{1}{{(1-\beta )c}_{1}}(ln(1+\frac{c_{1}{|s(0)|}^{1-\beta }}{c_{2}})) \end{array}$$ (16)

## SynRM controller design

### Design of extended state observer

The mathematical model of SynRM does not completely express the system due to unmodeled dynamics and parameters. Furthermore, uncertainties and external disturbances are also present, therefore error consistently exists between the actual and apparent model. Taking into account the effect of uncertainties, the motion equation of SynRM is expressed as follows:

$$\begin{array}{c} {\dot{\omega }}_{r}=\left(\psi +\Delta \psi \right)U-\left(B_{r}+\Delta B_{r}\right)\omega_{r}-\left(\delta +\Delta \delta \right)T_{L} \end{array}$$ (17)

Here $U=i_{q}^{*}$ is the control input, ;*ψ* = 3*P*^2^*φ*/2*J*;*B*_*r*_ = *B*/*J*, *δ* = *P*/*J* are nominal values. The values of motor parameter variations are represented as Δ*ψ*, Δ*B*_*r*_, and Δ*δ*. The disturbance in load *σ* is observed by an extended state observer and forwarded to get optimum controller performance, *γ* is calculated by adaptive gain law. *σ* and *γ* are represented as,

$$\begin{array}{cc} \gamma & =\Delta \psi U-\Delta B_{r}\omega_{r}-\Delta \delta T_{L} \end{array}$$ (18)

$$\begin{array}{cc} \sigma & =\psi \left(i_{q}-U\right)-{B_{r}\omega }_{r}-\delta T_{L} \end{array}$$ (19)

Then, (17) can be rewritten as follows:

$$\begin{array}{c} {\dot{\omega }}_{r}=\psi U+\gamma +\sigma \end{array}$$ (20)

Consider the system state equation is represented by:

$$\left\{\begin{array}{ll} {\dot{x}}_{1}\left(t\right) & =f\left(x,w,U\right)=x_{2}\left(t\right)+a_{1}U\\ {\dot{x}}_{2}\left(t\right) & =w(t) \end{array}\right.$$ (21)

$$\begin{array}{c} y=x_{1}\left(t\right) \end{array}$$ (22)

In (21), *x*_2_(*t*) is the disturbance input, while U is the control input, the disturbance is a bounded function, *a*_1_ is a positive constant, and *x*_1_(*t*) is equal to *ω*_*r*_(*t*). By using (20)-(22), the ESO for SynRM speed control is obtained. The ESO is based on the hyperbolic tangent function.

$$\begin{array}{c} \left\{ \begin{array}{cc} \epsilon_{1}\left(t\right) & =r_{1}\left(t\right)-\omega_{r}\left(t\right)\\ {\dot{r}}_{1}\left(t\right) & =r_{2}\left(t\right)+\gamma +\psi U-\rho_{1}\epsilon_{1}\left(t\right)\\ {\dot{r}}_{2}\left(t\right) & ={-\rho }_{2}tanh(\rho_{3}\epsilon_{1}\left(t\right)) \end{array}\right. \end{array}$$ (23)

The measured rotor speed is *ω*_*r*_(*t*) and the speed feedback signal is *r*_1_(*t*). The load disturbance torque *σ* is realized by *r*_2_. By using the parameter section principle discussed in [34], *ρ*_1_, *ρ*_2_, and *ρ*_3_ can be selected to satisfy *ρ*_1_, *ρ*_2_, *ρ*_3_ > 0.

### Disturbance-rejection sliding mode controller

The controller using advanced reaching law-based sliding mode control with ESO is named the disturbance-rejection sliding mode controller (DRSMC). To achieve the desired value of the state, the error between the reference and the actual speed is defined as:

$$\begin{array}{c} e_{1}=\;\omega_{r}^{*}-\omega_{r} \end{array}$$ (24)

$\omega_{r}^{*}$ is the desired speed reference command, *ω*_*r*_ is the output rotor speed. The derivative of the speed-tracking error is:

$$\begin{array}{c} {\dot{e}}_{1}=\;{\dot{\omega }}_{r}^{*}-{\dot{\omega }}_{r} \end{array}$$ (25)

putting the value of ${\dot{\omega }}_{r}$ from (20) gives,

$$\begin{array}{c} {\dot{e}}_{1}=\;{\dot{\omega }}_{r}^{*}-\psi U-\;\gamma -\sigma \end{array}$$ (26)

The important part of SMC is the appropriate selection of sliding manifold. In this paper, the sliding mode control and integral sliding mode control technique have been used. As one control input is used so one sliding surface *s* is required, for sliding mode control *s* selected as:

$$\begin{array}{c} s=k_{1}e_{1} \end{array}$$ (27)

*k*_1_ is the positive constant value, taking the derivative gives:

$$\begin{array}{c} \dot{s}=k_{1}{\dot{e}}_{1} \end{array}$$ (28)

using the value of ${\dot{e}}_{1}$ from (26):

$$\begin{array}{c} \dot{s}=k_{1}\left({\dot{\omega }}_{r}^{*}-\psi U-\;\gamma -\sigma \right) \end{array}$$ (29)

The proposed reaching law in (10) is also equal to $\dot{s}$, combining (10) and (29) gives:

$$\begin{array}{c} -c_{1}{\left|s\right|}^{\alpha sgn(|s|-1)}s\;-\;\frac{c_{2}{\left|s\right|}^{\beta }sgn\;(s)}{\left(1+Q|x|\right)}=k_{1}\left({\dot{\omega }}_{r}^{*}-\psi U-\;\gamma -\sigma \right) \end{array}$$ (30)

The speed controller block generates the control inputs signal, which is the q-axis reference current U, represented as:

$$\begin{array}{c} U=\;\frac{1}{k_{1}\psi }[{\dot{\omega }}_{r}^{*}-\hat{\gamma }-\sigma +c_{1}{\left|s\right|}^{\alpha sgn(|s|-1)}s\;+\;\frac{c_{2}{\left|s\right|}^{\beta }sgn(s)}{\left(1+Q|x|\right)}] \end{array}$$ (31)

The term −*σ*/*k*_1_*ψ* denotes the feed-forward compensation portion of ESO. The estimated parameter uncertainty value is $\hat{\gamma }$, which is modified according to the adaptive law given below

$$\begin{array}{c} \dot{\hat{\gamma }}=-B_{r}s\;,B_{r}>0 \end{array}$$ (32)

### Stability analysis of DRSMC

The system states are converged to zero in a finite time on the sliding surface undergoing the ASMCRL. The error of parameter estimation is defined as $\tilde{\gamma }=\gamma -\hat{\gamma }$ and $\dot{\tilde{\gamma }}=-\dot{\hat{\gamma }}$. To prove the stability of the DRSMC strategy, the Lyapunov function is chosen:

$$\begin{array}{cc} V & =\frac{1}{2}s^{2}+\frac{1}{2}\frac{k_{1}}{B_{r}}{\tilde{\gamma }}^{2} \end{array}$$ (33)

$$\begin{array}{cc} \dot{V} & =s\dot{s}+\frac{k_{1}}{B_{r}}\tilde{\gamma }\dot{\tilde{\gamma }} \end{array}$$ (34)

Using the values from (26) and (28)

$$\begin{array}{cc} \dot{V} & =s\left(k_{1}{\dot{e}}_{1}\right)+\frac{k_{1}}{B_{r}}\left(\gamma -\hat{\gamma }\right)\left(-\dot{\hat{\gamma }}\right) \end{array}$$ (35)

$$\begin{array}{cc} & =sk_{1}\left({\dot{\omega }}_{r}^{*}-\psi U-\;\gamma -\sigma \right)+\frac{k_{1}}{B_{r}}\left(\gamma -\hat{\gamma }\right)\left(B_{r}s\right) \end{array}$$ (36)

To make $\dot{V}$ negative definite and fulfill the Lyapunov stability criterion, the term $k_{1}s\dot{s}$ is added and subtracted, $\dot{s}$ value is taken from (10).

$$\begin{array}{cc} \dot{V} & =-sk_{1}(c_{1}{\left|s\right|}^{\alpha sgn(|s|-1)}s+\;\frac{c_{2}{\left|s\right|}^{\beta }sgn\left(s\right)}{\left(1+Q|x|\right)}\;)+sk_{1}({\dot{\omega }}_{r}^{*}-\psi U-\;\hat{\gamma }-\sigma +\\ & \;c_{1}{\left|s\right|}^{\alpha sgn(|s|-1)}s+\;\frac{c_{2}{\left|s\right|}^{\beta }sgn\left(s\right)}{\left(1+Q|x|\right)})-sk_{1}\left(\tilde{\gamma }\right)+sk_{1}\left(\gamma -\hat{\gamma }\right) \end{array}$$ (37)

$$\begin{array}{cc} & =-sk_{1}(c_{1}{\left|s\right|}^{\alpha sgn(|s|-1)}s+\;\frac{c_{2}{\left|s\right|}^{\beta }sgn\left(s\right)}{\left(1+Q|x|\right)}\;)+s\left(-k_{1}\psi U+k_{1}\psi U\right) \end{array}$$ (38)

$$\begin{array}{cc} & =-sk_{1}(c_{1}{\left|s\right|}^{\alpha sgn(|s|-1)}s+\;\frac{c_{2}{\left|s\right|}^{\beta }sgn\left(s\right)}{\left(1+Q|x|\right)}\;)\leq 0 \end{array}$$ (39)

The stability is guaranteed if the Lyapunov stability criterion is met i.e. $\dot{V}\leq 0$, hence the stability requirements are fulfilled, and the error will be zero in a finite time when the *k*_1_ > 0, *Q* > 0, *c*_1_ > 0, *c*_2_ > 0, 0 < *α* < 1, 0 < *β* < 1.

### Disturbance-rejection integral sliding mode speed regulator

The controller using advanced reaching law-based integral sliding mode control along with ESO has been named disturbance-rejection sliding mode speed regulator (DRSMSR). The ISMC technique has better steady-state performance as compared to the conventional SMC [35]. It reduces the steady state error by introducing the integration of error in the sliding surface. ISMC is used to propose the disturbance rejection sliding mode speed controller or regulator (DRSMSR). The integration of (24) is:

$$\begin{array}{c} e_{2}=\;\int \left(\omega_{r}^{*}-\omega_{r}\right)dt \end{array}$$ (40)

The sliding surface for ISMC is defined as:

$$\begin{array}{c} s=k_{1}e_{1}+k_{2}e_{2} \end{array}$$ (41)

*k*_1_ and *k*_2_ are positive constants. The derivative of sliding surface:

$$\begin{array}{c} \dot{s}=k_{1}{\dot{e}}_{1}+k_{2}{\dot{e}}_{2} \end{array}$$ (42)

where the derivative of *e*_2_ is equal to *e*_1_:

$$\begin{array}{c} {\dot{e}}_{2}=\;\left(\omega_{r}^{*}-\omega_{r}\right)=\;e_{1} \end{array}$$ (43)

Using the value of ${\dot{e}}_{1}$ from (26) and ${\dot{e}}_{2}$ from (43):

$$\begin{array}{c} \dot{s}=k_{1}\left({\dot{\omega }}_{r}^{*}-\psi U-\;\gamma -\sigma \right)+k_{2}e_{1} \end{array}$$ (44)

Using the proposed ASMCRL from (10) gives:

$$\begin{array}{c} -c_{1}{\left|s\right|}^{\alpha sgn(|s|-1)}s\;-\;\frac{c_{2}{\left|s\right|}^{\beta }sgn\;(s)}{\left(1+Q|x|\right)}=k_{1}\left({\dot{\omega }}_{r}^{*}-\psi U-\;\gamma -\sigma \right)+k_{2}e_{1} \end{array}$$ (45)

The estimated parameter uncertainty value is $\hat{\gamma }$, the control input *U* is obtained by solving (45):

$$\begin{array}{c} U=\;\frac{1}{k_{1}\psi }[{\dot{\omega }}_{r}^{*}-\hat{\gamma }-\sigma +k_{2}e_{1}+c_{1}{\left|s\right|}^{\alpha sgn(|s|-1)}s\;+\;\frac{c_{2}{\left|s\right|}^{\beta }sgn(s)}{\left(1+Q|x|\right)}] \end{array}$$ (46)

### Stability analysis of DRSMSR

For the stability analysis of the DRSMSR strategy, the Lyapunov function is chosen:

$$\begin{array}{cc} V & =\frac{1}{2}s^{2}+\frac{1}{2}\frac{k_{1}}{B_{r}}{\tilde{\gamma }}^{2} \end{array}$$ (47)

$$\begin{array}{cc} \dot{V} & =s\dot{s}+\frac{k_{1}}{B_{r}}\tilde{\gamma }\dot{\tilde{\gamma }} \end{array}$$ (48)

Using the values from (42) and (44)

$$\begin{array}{cc} \dot{V} & =s\left(k_{1}{\dot{e}}_{1}+k_{2}{\dot{e}}_{2}\right)+\frac{k_{1}}{B_{r}}\left(\gamma -\hat{\gamma }\right)\left(-\dot{\hat{\gamma }}\right) \end{array}$$ (49)

$$\begin{array}{cc} & =s\left(k_{1}\left({\dot{\omega }}_{r}^{*}-\psi U-\;\gamma -\sigma \right)+k_{2}e_{1}\right)+\frac{k_{1}}{B_{r}}\left(\gamma -\hat{\gamma }\right)\left(B_{r}s\right) \end{array}$$ (50)

To make $\dot{V}$ negative definite and fulfill the Lyapunov stability criterion, the term $k_{1}s\dot{s}$ is added and subtracted, $\dot{s}$ value is taken from (10).

$$\begin{array}{cc} \dot{V} & =-sk_{1}(c_{1}{\left|s\right|}^{\alpha sgn(|s|-1)}s+\;\frac{c_{2}{\left|s\right|}^{\beta }sgn\left(s\right)}{\left(1+Q|x|\right)}\;)+sk_{1}({\dot{\omega }}_{r}^{*}-\psi U-\;\hat{\gamma }-\sigma +\\ & \;k_{2}e_{1}+c_{1}{\left|s\right|}^{\alpha sgn(|s|-1)}s+\;\frac{c_{2}{\left|s\right|}^{\beta }sgn\left(s\right)}{\left(1+Q|x|\right)})-sk_{1}\left(\tilde{\gamma }\right)+sk_{1}\left(\gamma -\hat{\gamma }\right) \end{array}$$ (51)

$$\begin{array}{cc} & =-sk_{1}(c_{1}{\left|s\right|}^{\alpha sgn(|s|-1)}s+\;\frac{c_{2}{\left|s\right|}^{\beta }sgn\left(s\right)}{\left(1+Q|x|\right)}\;)+s\left(-k_{1}\psi U+k_{1}\psi U\right) \end{array}$$ (52)

$$\begin{array}{cc} & =-sk_{1}(c_{1}{\left|s\right|}^{\alpha sgn(|s|-1)}s+\;\frac{c_{2}{\left|s\right|}^{\beta }sgn\left(s\right)}{\left(1+Q|x|\right)}\;)\leq 0 \end{array}$$ (53)

It is clear that the system is globally stable and the error will converge to zero in a finite time. *k*_1_, *Q*, *c*_1_ and *c*_2_ are design parameters with any constant positive value, *α* and *β* are positive constant values mostly range between 0 and 1.

## Simulation verification

The effectiveness of ASMCRL and DRSMSR is discussed in this section using MATLAB simulation verification. The simulation of SynRM speed control using both proposed controllers DRSMC and DRSMSR is carried out. Four different cases of simulations have been carried out as described in Table 3. The results of the first two cases are compared with the PI speed regulator (PISR) to verify the effectiveness of the proposed reaching law and disturbance rejection capability. In case 3, DRSMSR is compared with DRSMC to verify reduced steady-state error and effectiveness against load torque variation. In case 4, DRSMSR is compared with the state-of-the-art adaptive nonsingular finite-time terminal sliding mode control for SynRM (ANFTSMC) presented in [20] to verify the fast dynamic response.

**Table 3:** Description of simulation scheme.

Speed reference (rpm)	Load	Validation
Case 1: 500	No Load Torque	Reaching Law
Case 2: 0 to 1010	Disturbance Fig 9a	Robustness
Case 3: 700	Load Torque Fig 9b	Lower Speed Error
Case 4: 800	Constant Load Torque 10 Nm	Faster Response Time

**Fig 3:**
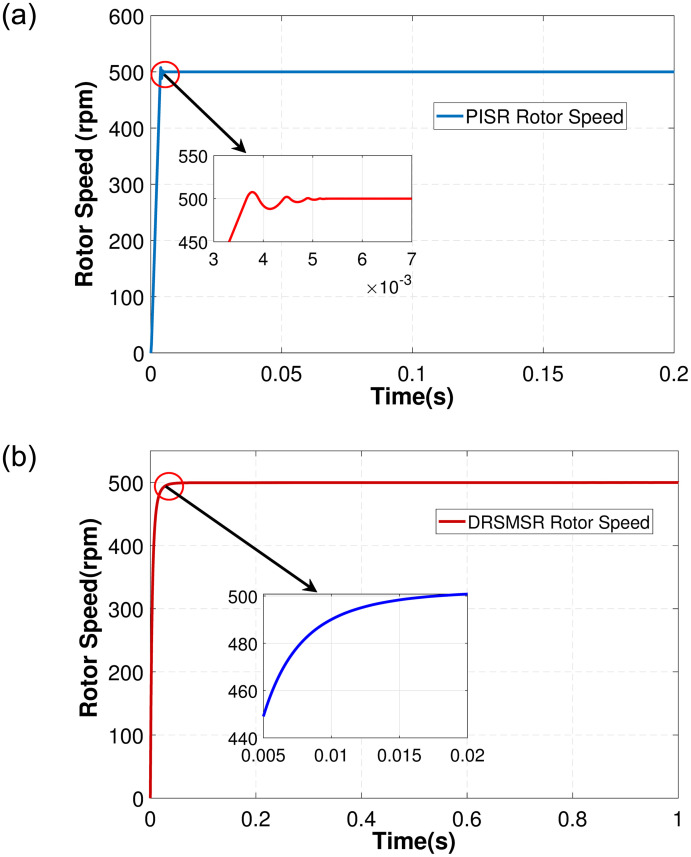
Simulation result of case 1 rotor speed. (a) PISR rotor speed, (b) DRSMSR rotor speed.

**Fig 4:**
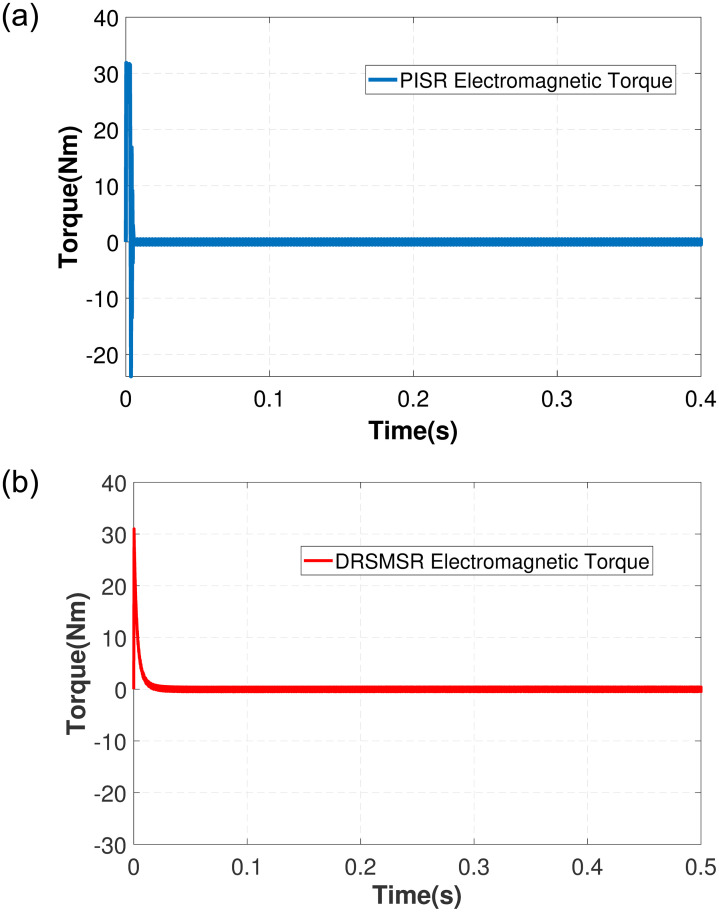
Simulation result of case 1 electromagnetic torque. (a) PISR electromagnetic torque, (b) DRSMSR electromagnetic torque.

**Fig 5:**
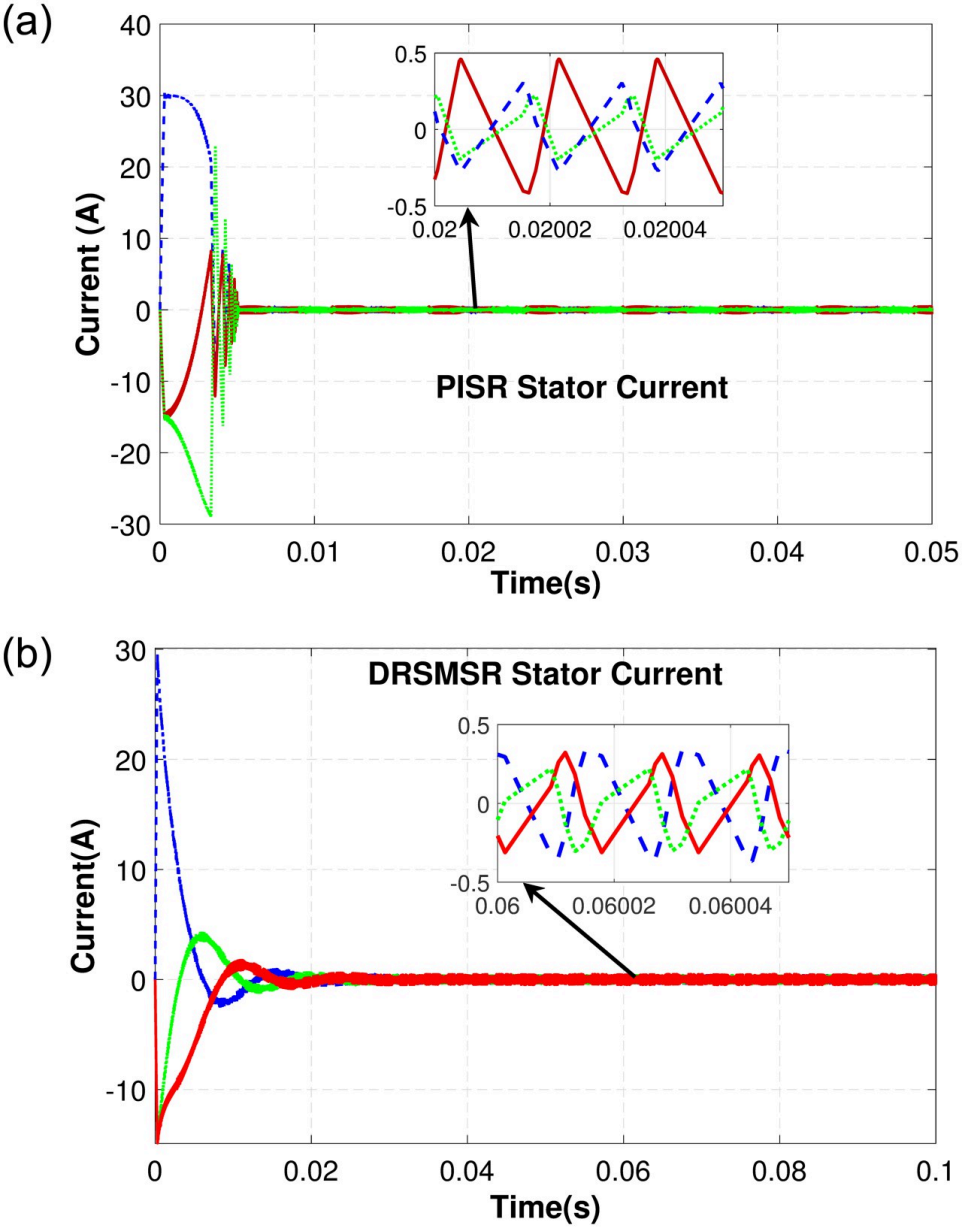
Simulation result of case 1 three-phase stator current. (a) PISR stator current, (b) DRSMSR stator current.

In Case 1, an input speed reference of 500rpm with no load torque is applied to PISR and DRSMSR and the results are shown in Figs 3–5. In this experiment, smooth reaching and chattering near the sliding surface have been observed. The results of case 1 rotor speed have been shown in Fig 3a and 3b. It is clear from Fig 3a that PISR has ripples in the output rotor speed. While using DRSMSR, the output rotor speed reaches the desired speed in a very smooth way. The reason for this improvement is due to the use of advanced reaching law for sliding mode control. The ASMCRL uses an adaptive gain with a higher value when the system state is far from the sliding surface and a lower value near the sliding surface to reduce chattering. Fig 3b shows that the reaching quality of DRSMSR near steady state value is far better as compared to the conventional PI controller in Fig 3a. It shows the effectiveness of the proposed reaching law. The torque ripples have also been reduced by the proposed reaching law as shown in Fig 4. The three-phase stator current comparison is shown in Fig 5.

**Fig 6:**
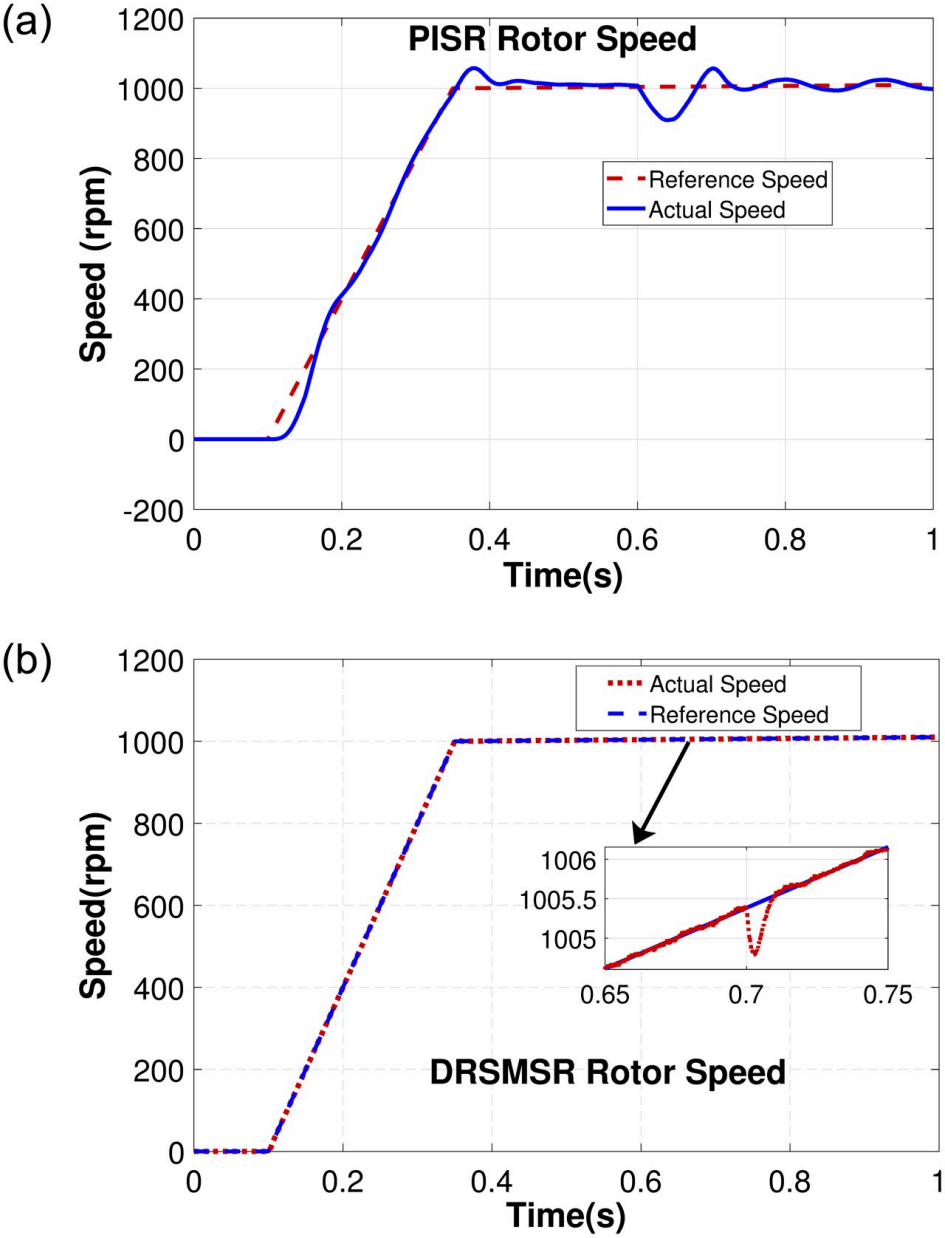
Simulation result of case 2 rotor speed. (a) PISR rotor speed, (b) DRSMSR rotor speed.

**Fig 7:**
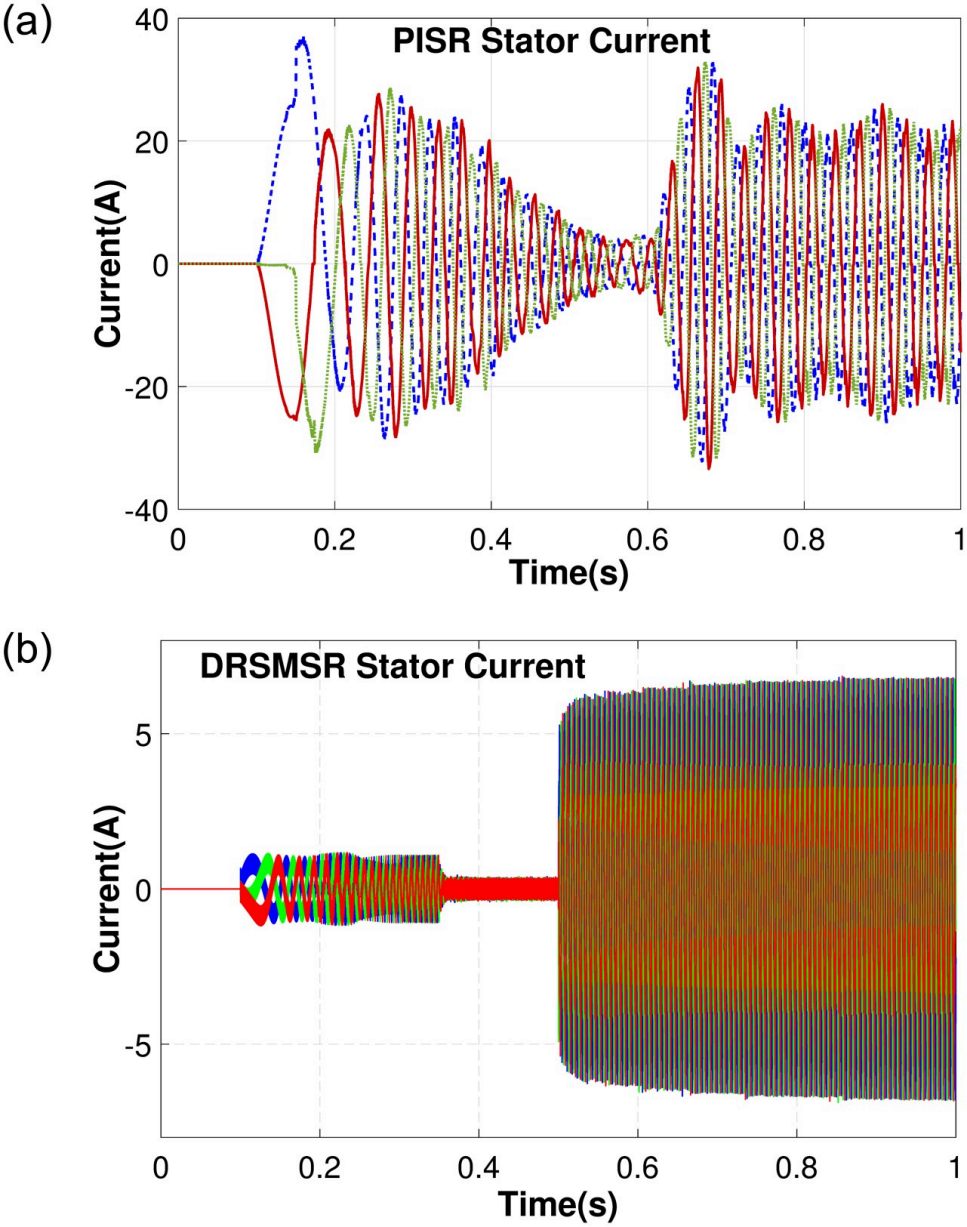
DRSMSR stator current. (a) PISR stator current, (b) DRSMSR stator current.

**Fig 8:**
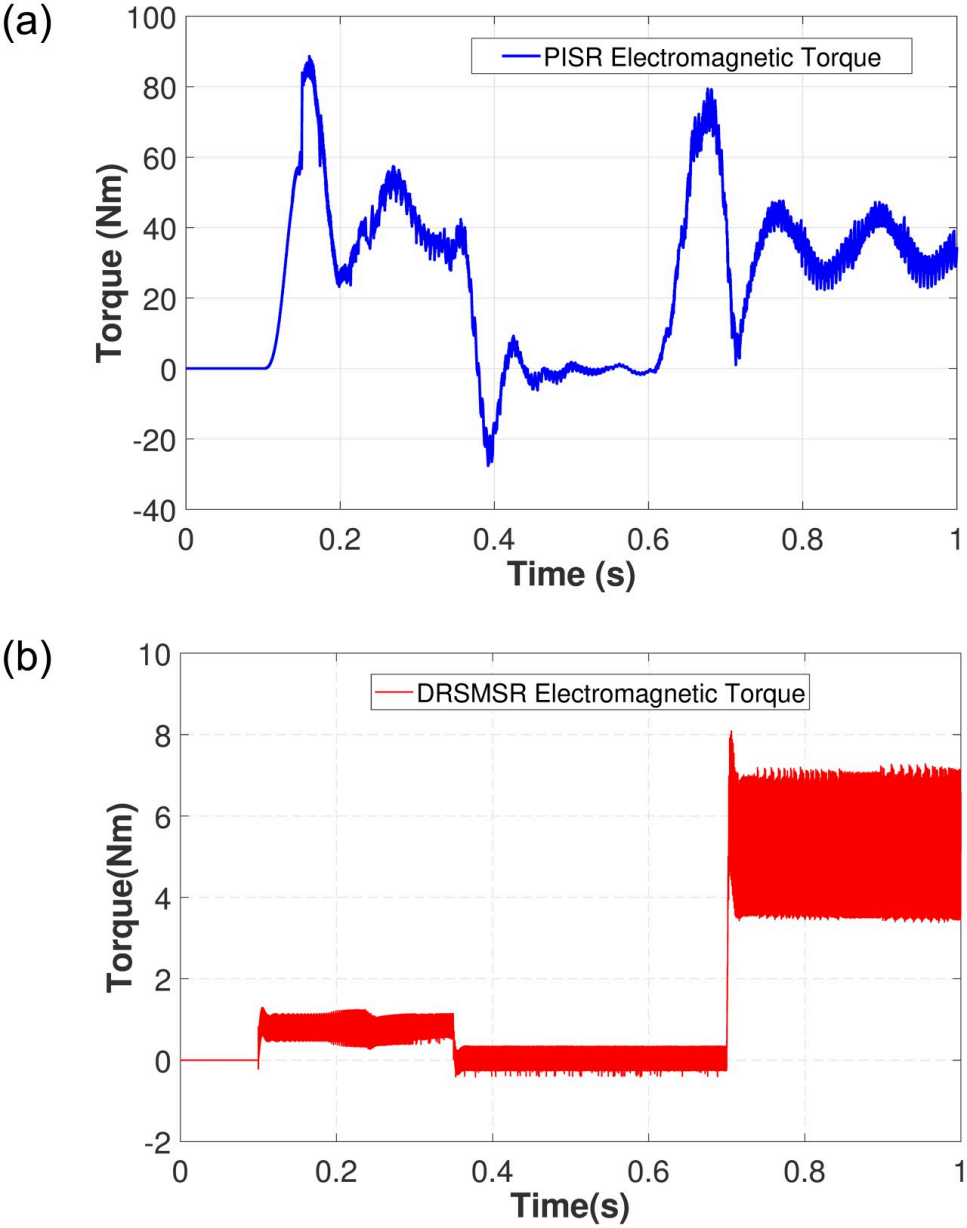
Simulation result of case 2 electromagnetic torque. (a) PISR electromagnetic torque, (b) DRSMSR electromagnetic torque.

**Fig 9:**
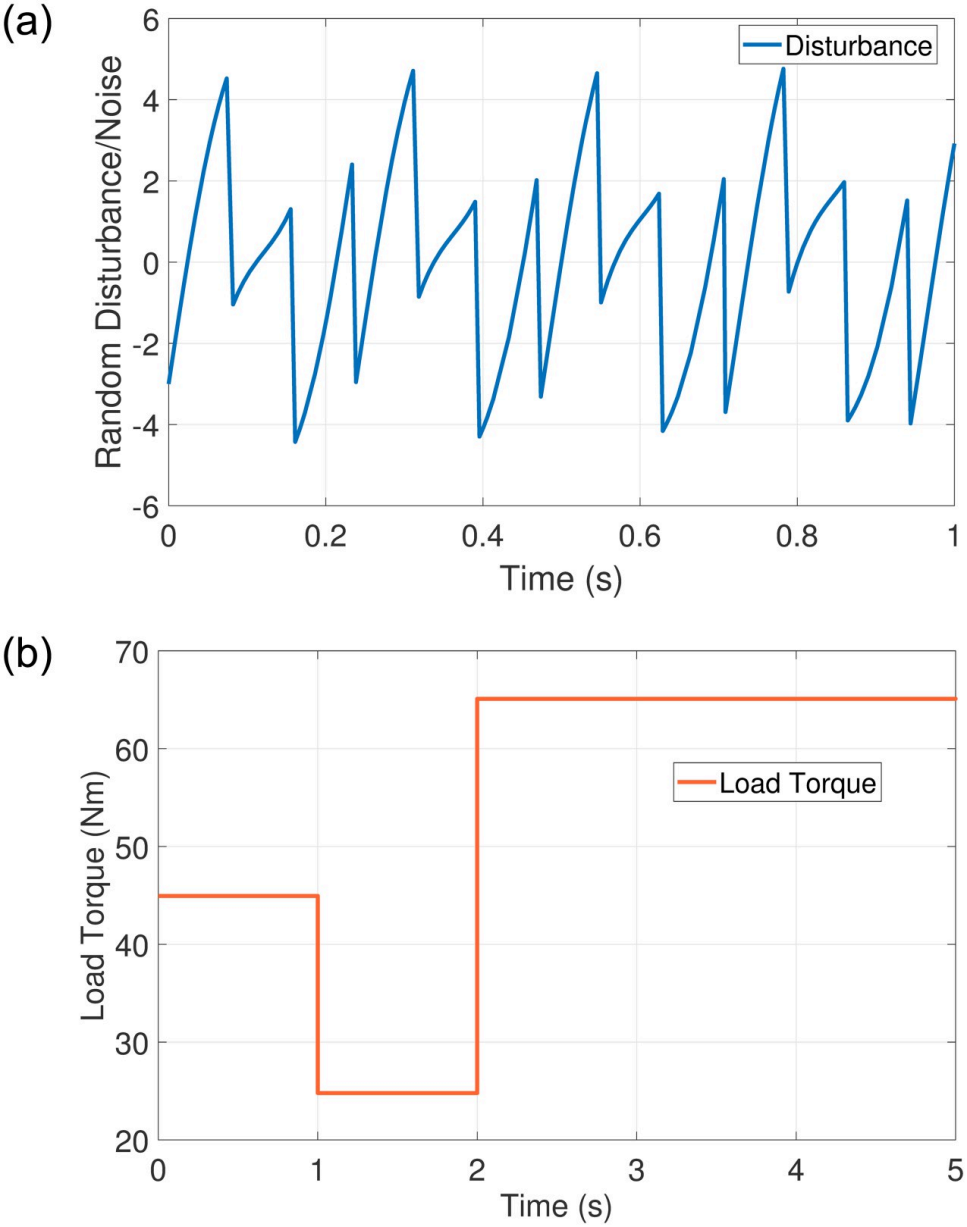
Disturbance and Load Torque profile. (a) Random disturbance in case 2, (b) Load Torque Profile in case 3.

Case 2 demonstrates the robustness of the proposed scheme against disturbance and load torque as shown in Figs 6–8. The random disturbance/noise (Gaussian) signal with mean = 0 and variance = 6 shown in Fig 9a, and a load torque of 5Nm at 0.7s are applied to SynRM. The input speed reference is varied starting from 0 to 1010 rpm. Fig 6 shows the rotor speed in the presence of disturbance and load torque. In Fig 6a, PISR has a visible error between the input reference and output rotor speed due to disturbance, while Fig 6b demonstrates that DRSMSR is robust against disturbance. The decrease in rotor speed at 0.7 seconds in Fig 6b is due to the load torque. The current and torque response of both DRSMSR and PISR are presented in Figs 7 and 8 respectively. The proposed scheme has a fast torque response. The disturbance rejection property of the proposed method is successfully validated in this case. The effect of disturbance and load torque is negligible, the reason for this satisfactory result is due to extended state observer-based integral sliding mode control. The proposed DRSMSR is proven to be robust against applied disturbances.

**Fig 10:**
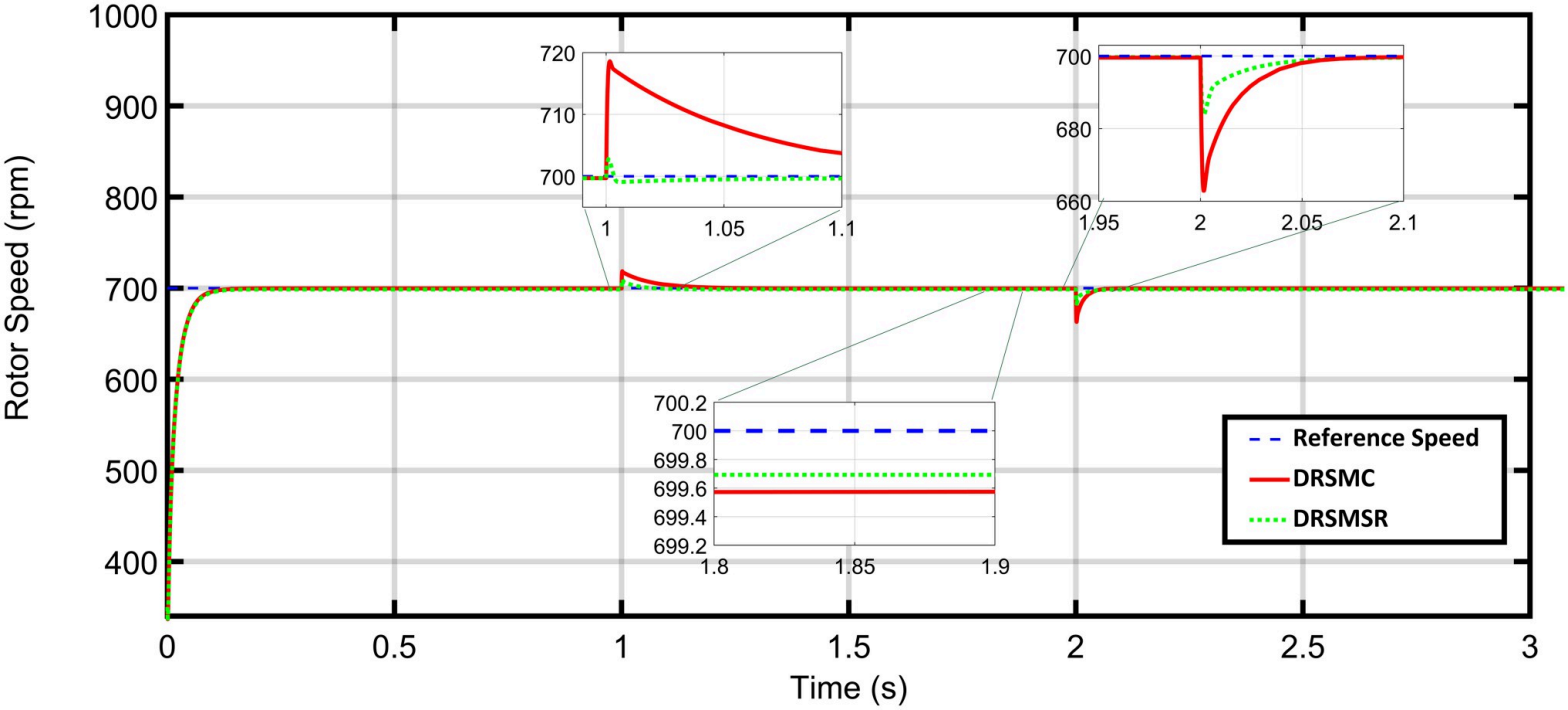
Simulation result of case 3 rotor speed (DRSMSR vs DRSMC).

In case 3, another experiment is carried out with increased load torque using DRSMSR and DRSMC. The load torque profile is shown in Fig 9b. The reference speed and the resultant rotor speed of DRSMC and DRSMSR are shown in Fig 10. The DRSMC has a maximum speed error of about 38.8rpm, while the proposed DRSMSR has a 19.81rpm. The steady-state error of DRSMSR is also less as compared to DRSMC. The improved results are due to the integral sliding mode controller used in DRSMSR.

**Fig 11:**
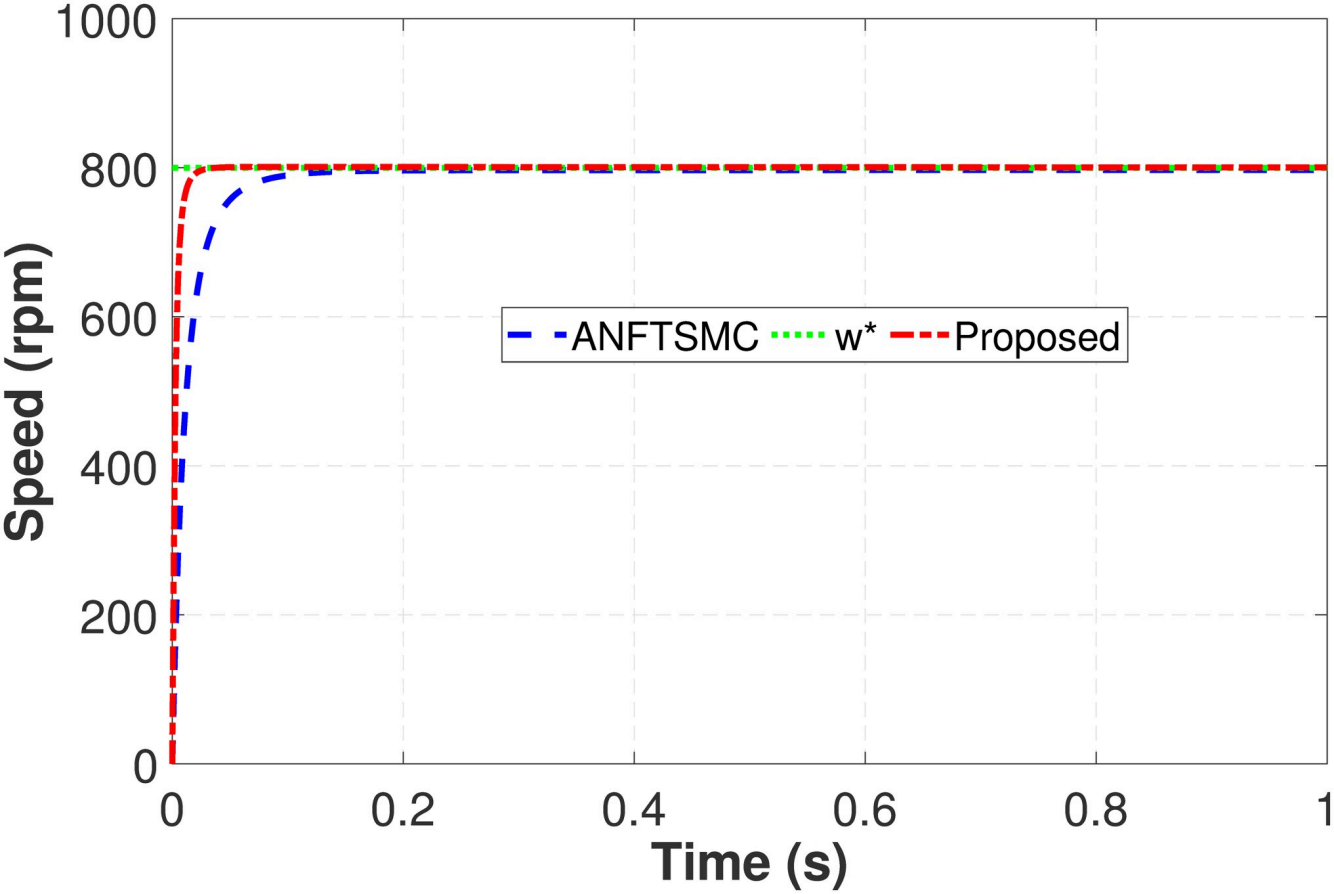
Simulation result of case 4 rotor speed (DRSMSR vs ANFTSMC).

**Fig 12:**
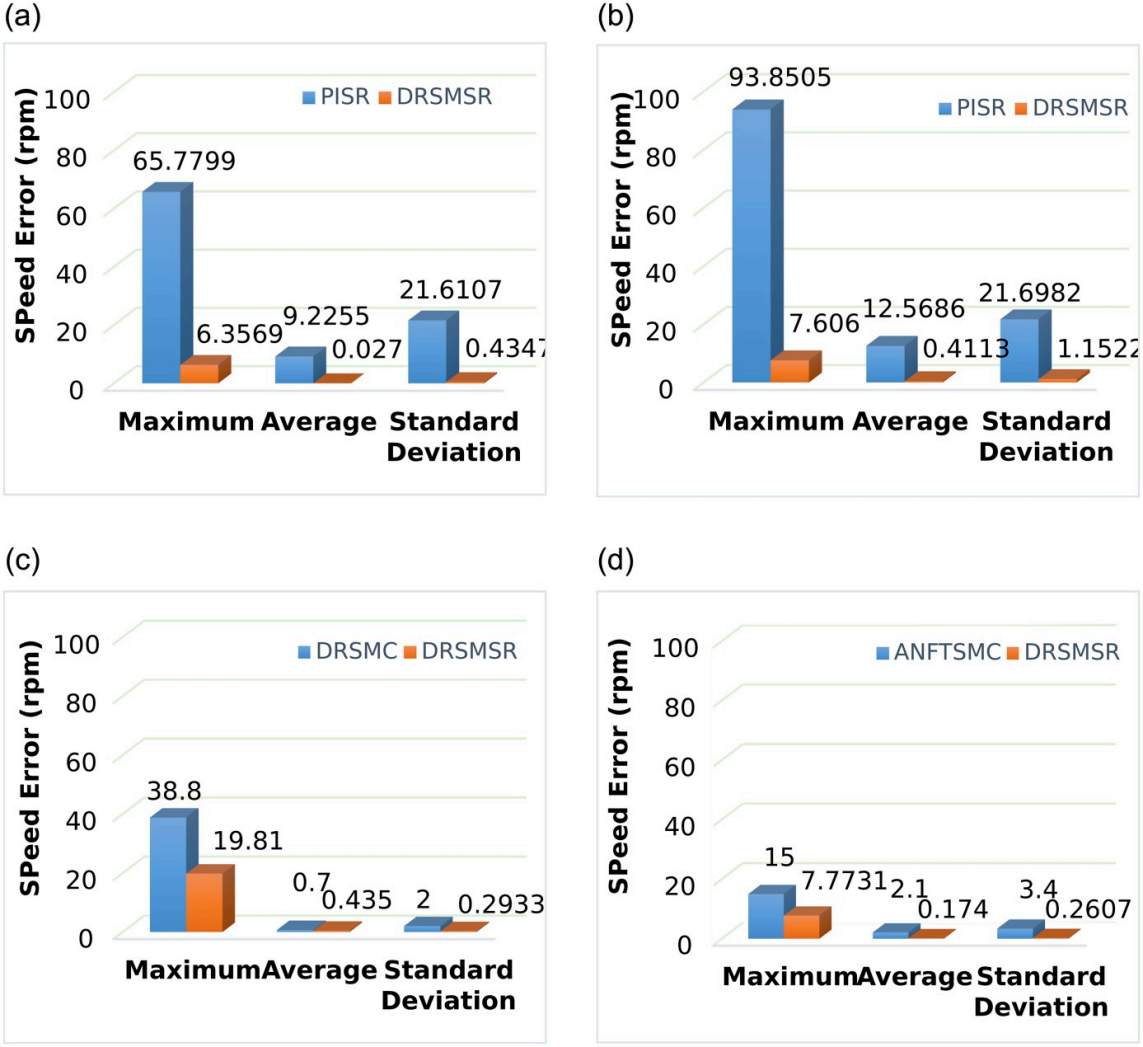
Performance analysis of SynRM rotor speed. (a) Case 1 (DRSMSR vs PISR) (b) Case 2 (DRSMSR vs PISR) (c) Case 3 (DRSMSR vs DRSMC) (d) Case 4 (DRSMSR vs ANFTSMC).

Lastly, in case 4 to verify the faster dynamic response DRSMSR is compared with ANFTSMC, as shown in Fig 11. The time required for reaching a reference speed of 800 rpm is 0.221s in ANFTSMC. While the proposed scheme uses only 0.05s. The response of the proposed technique is 22.62% faster as compared to ANFTSMC. This is achieved with the help of the proposed advanced reaching law, which provides a faster response with no overshoot. The disturbance rejection property of the proposed scheme reduces the maximum speed error as shown in Fig 12.

These four cases verify that the proposed scheme has less speed error in case of load torque and external disturbance. It is a robust control technique with a fast dynamic response and less speed error. The speed tracking error is reduced as compared to the conventional PI controller, DRSMC and ANFTSMC. The average and standard deviation of tracking errors is better in the case of DRSMSR. In case 1, the maximum speed error is 65.77rpm with PISR, while DRSMSR has a maximum error of 6.3569rpm. The improvement is due to advanced reaching law. The average error is 9.22rpm and 0.027rpm, while the standard deviation is 21.61 and 0.4347rpm for PISR and DRSMSR, respectively. In case 2, the maximum speed error is 93.85rpm with PISR, while DRSMSR has a maximum error of 7.606rpm. The high value of speed error in PISR is due to disturbances. The average error is 12.56rpm and 0.4113rpm, while the standard deviation is 21.69 and 1.15rpm for PISR and DRSMSR, respectively. In case 3, the maximum speed error is 38.8rpm and 19.81rpm, the average error is 0.7rpm and 0.435rpm, while the standard deviation is 2 and 0.293rpm for DRSMC and DRSMSR, respectively. In case 4, the maximum speed error is 15rpm and 7.773rpm, the average error is 2.1rpm and 0.174rpm, while the standard deviation is 3.4rpm and 0.2607rpm for ANFTSMC and DRSMSR, respectively.

**Fig 13:**
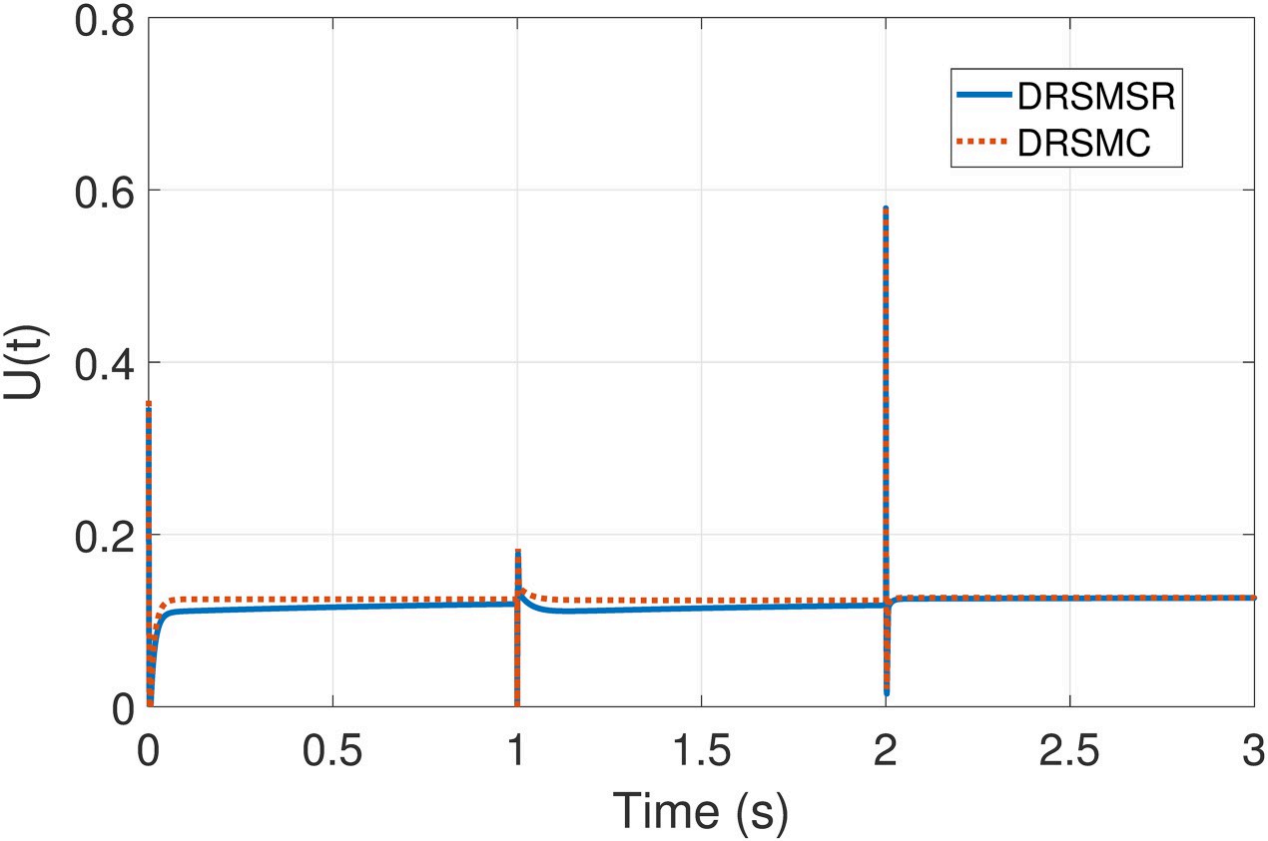
Simulation result of control input (DRSMSR vs DRSMC).

The simulation results using four different cases have been discussed. The comparison between the proposed DRSMSR with the typical PI controller, DRSMC, and state-of-the-art ANFTSMC has been presented. It is evident from the results that DRSMSR has better overall performance, during the loading process, the dynamic response is very fast, with less steady-state error and speed tracking is satisfactory. Furthermore, by using the advanced reaching law, the chattering has been reduced. The control inputs of DRSMSR and DRSMC are shown in Fig 13.

The constants, gains, and design parameters of the controllers DRSMSR and DRSMC are selected using the trial and error method. Values of the gain parameters are as follows:

- *k*_1_ = 5, *k*_2_ = 2, *c*_1_ = 3000, *c*_2_ = 1500
- *α* = 0.8, *β* = 0.5, *Q* = 23
- *ρ*_1_ = *ρ*_2_ = 160, *ρ*_3_ = 0.85

## Conclusion

In this work, an advanced reaching law for sliding mode control for SynRM speed control is proposed. A nonlinear observer is also developed to efficiently eliminate the effect of uncertainties from the output. Based on the proposed reaching law and observer, two disturbance rejection sliding mode speed controllers, DRSMSR and DRSMC have been developed to attain the required control objectives. The DRSMSR has better performance as compared to DRSMC as it provides reduced steady-state error and a fast convergence rate. The DRSMSR is capable to be used in practical applications by replacing the typical PI controller. The proposed controllers use adaptive rate reaching law and extended state observer to detect and online compensate for the disturbances and generate current commands. By applying various speed commands under varying load torque and disturbance, the proposed system produces a satisfactory response. The robust system shows a faster dynamic response with reduced speed tracking errors. The overall performance of the proposed DRSMSR control scheme is satisfactory. The global stability of the proposed system is verified using Lyapunov stability analysis. In the future, the possibility of SynRM drive application in an electric vehicle with an appropriate control strategy may be studied. Furthermore, research on performance analysis of SynRM and different reference values of speed and torque can be carried out.
